# Associated factors for a false negative cardiovascular magnetic resonance perfusion study: a CE-MARC substudy

**DOI:** 10.1186/1532-429X-15-S1-P214

**Published:** 2013-01-30

**Authors:** Sven Plein, Ananth Kidambi, Steven Sourbron, Neil Maredia, Akhlaque Uddin, Manish Motwani, David P Ripley, Bernhard A Herzog, Julia Brown, Jane Nixon, Colin Everett, John P Greenwood

**Affiliations:** 1 Department of Cardiology, Multidisciplinary Cardiovascular Research Centre & Leeds Institute of Genetics, Health and Therapeutics, Leeds, UK; 2 Division of Medical Physics, University of Leeds, Leeds, UK; 3 Clinical Trials Research Unit, University of Leeds, Leeds, UK

## Background

Diagnosis of coronary ischemia by perfusion CMR has high sensitivity and specificity when using X-ray coronary angiography as the reference standard. A variety of possible reasons have been given for false negative perfusion CMR studies, such as suboptimal image quality, technical reasons, or the potential discrepancy between angiographic stenosis and detectable myocardial hypoperfusion. The rates at which these factors occur have not been specifically studied to date. The CE-MARC study prospectively enrolled 752 patients with suspected coronary artery disease, scheduled to undergo CMR, SPECT and X-ray coronary angiography [1]. We assessed potential reasons for the false negative CMR perfusion studies within CE-MARC.

## Methods

All patients with significant coronary stenosis (≥70% stenosis of a first order coronary artery ≥2mm diameter, or left main stem stenosis ≥50% as measured by quantitative coronary angiography (QCA)), who had a normal or probably normal CMR perfusion analysis from the original, blinded read were selected from the CE-MARC population. Patient and imaging characteristics were analyzed. Myocardial perfusion reserve (MPR) was calculated offline (PMI v0.4) from CMR stress and rest perfusion images using the Fermi model, with arterial input defined in the LV blood pool, and the whole mid-LV short axis myocardial slice used as tissue response.

## Results

**Figure 1:**
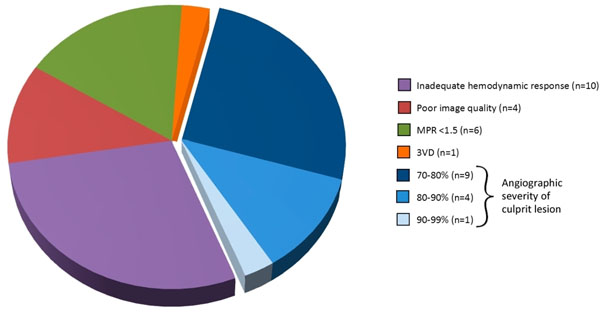
**Factors associated with a false negative CMR perfusion scan.** One factor is shown per patient (n=35).

36 patients with a false-negative CMR result were identified (Table 1). 1 patient had perfusion image quality recorded as "unusable," and was excluded from further analysis. 4 (11%) patients' images were graded as "poor quality." 10 patients (29%) had inadequate hemodynamic response to adenosine (SBP decrease <10mmHg or heart rate increase <10beats/min). 1 patient (3%) had angiographic 3-vessel disease, supporting balanced ischemia. A further 6 patients (17%) had an adequate hemodynamic response but MPR <1.5, suggesting possible inadequate vasodilatation (in the absence of triple vessel disease). Of the remaining 14 patients, mean QCA diameter of culprit stenoses was 74% ± 12%, close to the angiographic cut-off of ≥70% for significant disease (Figure 1).

**Table 1:** Patient characteristics for false negative perfusion CMR in CE-MARC.

	False negative patients	CE-MARC whole population
N	35	752
Age (years)	61 ± 7	60 ± 10
Male	29 (83%)	471 (63%)
Body-mass index (kg/m^2^)	28.3 ± 4.0	29.2 ± 4.4
Resting BP (mmHg)	125/71 ± 20/10	138 / 79 ± 21/11
LAD disease	19 (54%)	183 (25%)
Circumflex disease	16 (46%)	133 (18%)
RCA disease	8 (23%)	110 (15%)
Left main disease	2 (6%)	23 (3%)

Data as n (%) or mean ± SD.

## Conclusions

Of the multiple potential factors contributing to false negative CMR perfusion studies, over one third of false negative studies may have been related to lack of efficacy of pharmacological stress at the standard adenosine dose of 140 mcg/kg/min. A substantial proportion of patients had coronary stenosis severity close to the angiographic cut-off of 70%, which may represent discordance between anatomical and functional assessment. Non-diagnostic image quality and 3-vessel disease made a relatively small contribution to false-negative CMR studies.

## Funding

CE-MARC was funded by the British Heart Foundation (BHF). JPG and SP receive an educational research grant from Philips Healthcare. SP is funded by a BHF fellowship (FS/1062/28409).
